# Enhancing the tribopositive characteristics of polyvinyl alcohol (PVA)-carbon composites by optimizing the PVA-carbon interaction with various carbon fillers†

**DOI:** 10.1039/d4na00820k

**Published:** 2024-12-09

**Authors:** Jian Ye Cheong, Jason Soon Chye Koay, Sanjeev Raj Gopal, Thamil Selvi Velayutham, Wee Chen Gan

**Affiliations:** a Low Dimensional Materials Research Center, Department of Physics, Faculty of Science, Universiti Malaya 50603 Kuala Lumpur Malaysia t_selvi@um.edu.my; b Kelip-kelip! Center of Excellence for Light Enabling Technologies, School of Energy and Chemical Engineering, Xiamen University Malaysia Selangor Darul Ehsan 43900 Malaysia wcgan@xmu.edu.my; c College of Chemistry and Chemical Engineering, Xiamen University Xiamen 361005 China

## Abstract

Incorporating carbon-based fillers into triboelectric nanogenerators, TENGs, is a compelling strategy to enhance the power output. However, the lack of systematic studies comparing various carbon fillers and their impact on tribopositive contact layers necessitates further research. To address these concerns, various carbon fillers (including buckminsterfullerene (C_60_), graphene oxide (GO), reduced graphene oxide (rGO), multi-wall carbon nanotube (MWCNT), and super activated carbon (SAC)) with distinct structural and electrical properties are mixed with polyvinyl alcohol, PVA, to form PVA-carbon composites and used as tribopositive layers in the contact-separation of TENGs. The results show that PVA-SAC provides the largest enhancements to the electrical outputs of the TENG. At the optimal loading of 1 wt%, PVA-SAC composites yielded a peak power density of 12.8 W m^−2^, a substantial 220% enhancement compared to pristine PVA. The mechanism governing the enhancement is determined by analysing the changes in electrical and structural characteristics caused by the addition of various carbon fillers. Dielectric measurements indicated that enhanced dielectric properties did not significantly contribute to the observed increase in the triboelectric performance. Instead, Raman and FTIR analyses revealed a correlation between the PVA-carbon interactions and an increase in the D/G ratio of carbon fillers, accompanied by a reduction in hydrogen-bonded –OH groups within PVA. This suggests that the interaction between the π electrons of sp^2^ hybridized carbon atoms and the oxygen lone pairs in PVA inhibits hydrogen bond formation, leading to an increase in free –OH groups. Consequently, these free –OH groups enhanced the electron-donating capability and improved the tribopositive behaviour of the PVA-carbon composites. Our results proved that filler-matrix interactions are paramount in engineering high-performance TENGs by controlling the electron affinity of the triboelectric layers.

## Abstract 2

Carbon-based fillers in PVA reduce the hydrogen bonding and frees up –OH groups, enhancing its tribopositivity.

## Graphical abstract

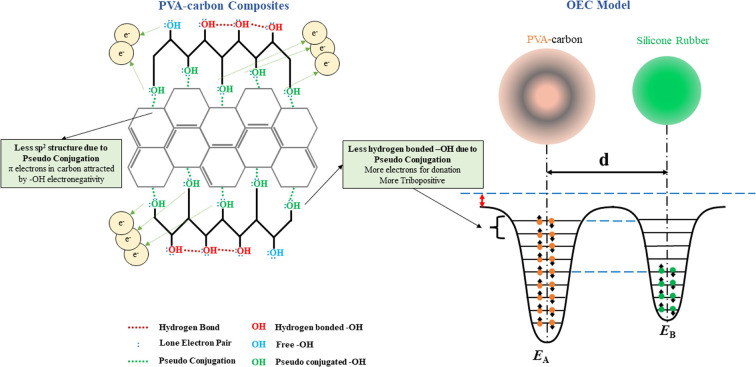

## 1 Introduction

The increment in the usage of smart miniature electronics in recent years has resulted in a need for a self-sustainable energy source.^1–7^ Triboelectric nanogenerators, TENGs, have emerged as a promising energy harvesting technology due to their simple structure and self-sustainable characteristics. Based on the coupling effects of contact electrification and electrostatic induction, TENGs can convert low-frequency ambient mechanical motion into electricity with high efficiency.^8–11^ Since the introduction of TENGs in 2012, multiple efforts have been carried out over the past decade to increase the surface charge density of TENGs, which include modification of surface morphology,^1,12–15^ chemical functionalization^16–20^ and compositing polymers with extrinsic materials.^21–25^ Among these methods, utilizing polymer composites as triboelectric materials is more favourable as it provides a stable enhancement in the TENGs' power output. In particular, carbon-based particles are effective additives to enhance the durability and power output of triboelectric layers^26–33^ due to their favourable properties such as high electrical conductivity and high charge storing capacity. The addition of carbon-based particles to polymers produces composites with these favourable properties, which enhance the power output of TENGs. Previous studies have proven that incorporating carbon black, CB into typical TENG polymers, such as polydimethylsiloxane, PDMS and tetra-polyurethane, TPU resulted in increased capacitance of the composites which contributes to the enhancements in TENG power outputs.^28,33^ Other than CB, there is a wide variety of carbon fillers which can also be utilized to improve the power outputs of TENGs. Investigations comparing the contributions of various carbon fillers towards the TENGs are important to ensure the optimal carbon is used as the polymer filler. Several analyses have been conducted in recent years to determine the ideal carbon filler for TENGs. A comparison between multiwalled carbon nanotube, MWCNT, reduced graphene oxide, rGO and CB as fillers in polyacrylonitrile, PAN nanofibers for the tribonegative layer in TENGs showed that MWCNT provided the largest enhancement in the power output of TENGs, while CB reduced the power output.^34^ The improvement contributed by MWCNT was attributed to the increased dielectric constant, whereas the reduction in power output was associated with the incompatibility of CB as a tribonegative material. Recently, it was discovered that PDMS composited with graphene oxide, GO provided larger enhancements in TENGs' outputs compared to graphite and rGO.^28^ The oxygen functional groups in GO interact with the PDMS polymer host, enabling a homogenous dispersion and thus higher electrical outputs. Besides, the lower electrical performances of graphite and rGO was linked to their higher conductivity compared to GO, resulting in higher leakage currents and lower effective power outputs.

While specific types of carbon have evidently improved the output of certain TENGs, there is a significant lack of understanding of the exact enhancement mechanism, which hinders a comprehensive and fundamental knowledge of the phenomenon. Currently, most studies incorporate carbon fillers into tribonegative polymers to enhance TENG performance. However, a significant question remains: can the same approach be applied to improve tribopositive materials? Given the relative scarcity of high-performance tribopositive options compared to tribonegative ones, exploring this avenue could be highly beneficial. Another notable research gap in the field of carbon-based TENGs is the lack of in-depth understanding of the mechanism governing the enhancement in the TENGs' power output. A systematic study, which includes a diversified range of carbon fillers with distinct morphologies, structural and electrical characteristics (such as 0D fullerenes, 1D carbon nanotubes, 2D graphene and 3D activated carbon), will provide valuable insights for a better understanding of the working principles behind the enhancements of the TENGs' performances. It is crucial to utilize a wider range of electrical and structural characterizations to analyse how the carbon fillers change the properties of the polymer matrix. Frequency-dependent dielectric and conductivity measurements would provide deeper insights into the distinct contributions of each carbon filler to the overall TENG performance, enabling targeted material design and optimization for specific applications. Structural aspects beyond simple filler composition also deserve consideration. Specific intermolecular interactions such as hydrogen bonding between the host polymer and carbon fillers, variations in crystallinity (such as amorphous, semi-crystalline and crystalline) and surface morphology features (such as roughness and porosity) could significantly influence TENG power output by affecting charge transfer, triboelectric generation, and other critical mechanisms. Investigating these factors in-depth can unlock new avenues for optimizing TENG performance.

To systematically investigate the impact of carbon fillers on TENG performance, a diverse range of carbon fillers, including C_60_, GO, rGO, MWCNTs, and SAC were incorporated into a PVA matrix for tribopositive layer of TENG. While all carbon fillers enhanced electrical output, our findings challenge the conventional perception that solely electrical properties dictate TENG performances. Instead, we unveil a novel mechanism where intricate structural interactions between the carbon fillers and PVA matrix significantly influence the electron-donating capacity of the composite, ultimately determining TENG efficiency. This discovery opens new avenues for optimizing TENG performance by manipulating filler-matrix interactions at the molecular level.

## 2 Experimental section

### 2.1 Materials

PVA (molecular weight of 145 000, fully hydrolysed) and SAC (specific surface area of 1300 m^2^ g^−1^) were purchased from US Research Nanomaterials, Inc. GO (specific surface area of 400 m^2^ g^−1^), rGO (specific surface area of 900 m^2^ g^−1^), and C_60_ fullerene (specific surface area of 1310 m^2^ g^−1^) were purchased from Sigma-Aldrich. MWCNT (specific surface area of 233 m^2^ g^−1^) was purchased from Galaxy Nanotech. Silicone rubber (Ecoflex 00-50) was purchased from smooth-On, Inc. The other reagents were commercially purchased, and all the materials were used as received.

### 2.2 Sample preparation

#### 2.2.1 Preparation of PVA and PVA-carbon as tribopositive material

1 g of PVA was dissolved in 10 ml of deionized water along with varying weight percentages (wt%) of carbon fillers, ranging from 0.25 wt% to 3.0 wt%. The solution stirring at 175 °C for 1 h to ensure complete dissolution of PVA and homogeneous distribution of carbon fillers. Subsequently, the mixture was cast into a Petri dish and dried in an oven at 43 °C overnight to evaporate the residual solvent, yielding a PVA-carbon composite film.

#### 2.2.2 Preparation of silicone rubber (SR) as tribonegative material

A 25 mm × 25 mm glass substrate was first coated with silver to serve as the current collector. Subsequently, copper strips with conductive adhesives were attached as extended electrodes. Next, the elastomer and curing agent of the silicone rubber were mixed at a 1 : 1 ratio and spin-coated on the glass substrates at 250 rpm for 60 s. The mixtures were left to cure at room temperature for 24 h.

#### 2.2.3 TENG assembly

Copper plates of 20 mm × 20 mm with an extended edge of 5 mm in width were prepared as the current collector as well as the substrate. The tribopositive PVA and PVA-carbon films were cut and attached to the substrates. Both the tribopositive PVA-carbons and the tribonegative SR were then attached to the electrodynamic shaker for the electrical output and power measurements.

### 2.3 Characterization and measurements

#### 2.3.1 Electrical output and power output measurement

The electrodes of the TENGs were connected to the terminals of a Keithley 6517B Electrometer. The electrodynamic shaker was controlled by a function generator to simulate a periodic contact-separation motion between the two triboelectric layers. The parameters of the motion were controlled to a contact force of 50 N, with an interspacing distance of 10 mm and a frequency of 1 Hz. The open-circuit voltage *V*_oc_, short-circuit current density *J*_sc_ and short-circuit charge density *Q*_sc_ of TENGs were then measured using the Keithley 6517B Electrometer. To characterize the power output, a range of resistive loads from 100 kΩ to 500 MΩ were connected to the TENG *via* a full-wave rectifier. The rectified current was measured, and power density, *P*, was calculated using the equation *P* = *I*^2^*R*/*A*, where *I* is the measured current, *R* is the load resistance, and *A* is the effective contacting surface area of the TENGs. To assess the charging capabilities of the TENGs, the voltage across capacitors ranging from 1 μF to 330 μF was monitored over time.

#### 2.3.2 Dielectric and conductivity measurements

PVA and PVA-carbon films were clamped in between two stainless-steel plates attached with electrode extensions and connected to Keysight E5061B impedance analyser. Sweep measurements were made on samples with frequencies ranging from 1 kHz to 30 MHz to obtain the capacitance, *C*_p_ and tangent loss, *D*. The dielectric constant was calculated using the equation *ε*′ = *C*_p_*d*/*ε*_0_*A*, where *C*_p_ is the measured capacitance, *d* is the thickness of the sample, *ε*_0_ is the dielectric permittivity of vacuum and *A* is the area of the stainless-steel plate. The dielectric loss was calculated using the equation *ε*′′ = *ε*′*D*, where *D* is the measured loss tangent. From the dielectric measurements, the real and imaginary conductivities were calculated using the equations σ* = iωε* (*σ*′ = *ωε*′′, *σ*′′ = *ωε*′), where *ω* = 2π*f*.

#### 2.3.3 Structural analyses

Raman spectroscopy was performed using Renishaw inVia Raman microscope, with 532 nm laser excitation source at 100% laser power and wavenumber 100 to 4000 cm^−1^. Temperature dependent FTIR measurements were carried out using PerkinElmer Frontier IR/NIR with wavenumber ranging from 650 cm^−1^ to 4000 cm^−1^, with a step size of 4 cm^−1^ and temperature ranging from 25 °C to 100 °C. Beer Lambert's law was applied to convert the FTIR results from transmittance % (*T*%) into absorbance (*A*) using the equation log(*T*%) = 2 − *A*. XRD measurement was conducted using Ultima-IV by Rigaku, with 2*θ* ranging from 5° to 80°. The surface morphologies of PVA-carbons were imaged *via* SEM using an electron microscope (FEI Quanta SEM Model 400F).

## 3 Results and discussion

**Fig. 1:**
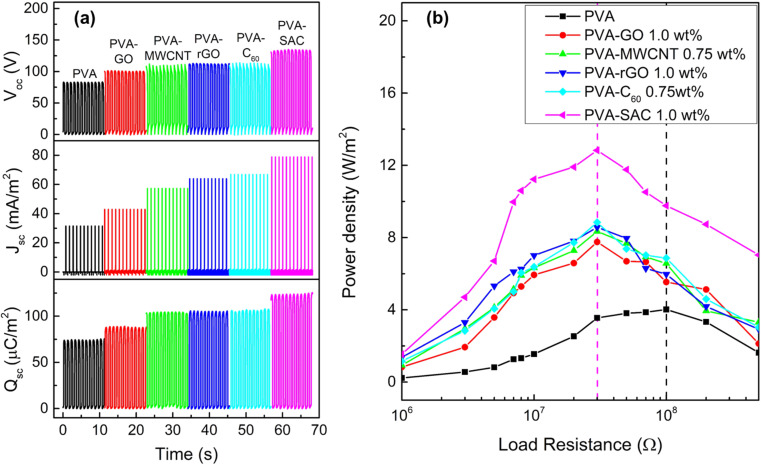
(a) Open circuit voltage (*V*_oc_), short-circuit current density (*J*_sc_) and short-circuit charge density (*Q*_sc_), and (b) power density curves of pristine PVA and each PVA-carbon at the optimal loading concentration.

The contact-separation TENGs in this study utilized PVA-carbon composites as the tribopositive layer and silicone rubber (SR) as the tribonegative layer. The TENG output characteristics of PVA composites incorporating different carbon filler loadings were investigated. Detailed performance metrics are provided in Fig. S1 and S2 of the ESI.† All carbon fillers demonstrably enhanced the electrical outputs of the TENGs. The electrical outputs increase with increasing loading wt% until reaching an optimal value, thereafter, experiencing a decline. Notably, C_60_ and MWCNT peaked at 0.75 wt%, while rGO, GO and SAC reached their optima at 1 wt%. Fig. 1 presents a comparison of the performance characteristics of pristine PVA and PVA-carbons at their respective optimal loading concentrations. Fig. 1(a) presents the individual metrics of open-circuit voltage (*V*_oc_), short-circuit current density (*J*_sc_), and short-circuit charge density (*Q*_sc_) for each material. The TENGs exhibited an increasing electrical output trend in the following order: pristine PVA < PVA-GO < PVA-MWCNT < PVA-rGO < PVA-C_60_ < PVA-SAC. The PVA-SAC/SR TENG incorporating 1 wt% SAC exhibited the maximum enhanced performance, generating *V*_oc_ of 135 V, *J*_sc_ of 79 mA m^−2^, and *Q*_sc_ of 125 μC m^−2^. These values represent increases of 65%, 150%, and 65%, respectively, compared to the pristine PVA/SR TENG. Fig. 1(b) shows the power densities of the TENGs as functions of various load resistances. The same rankings as their respective electrical outputs are observed, whereby the PVA-SAC/SR TENG with 1 wt%, which is the best-performing TENG, generated a maximum power density of 12.8 W m^−2^ at 30 MΩ. Compared to pristine PVA/SR TENG, the power density increased by 220%. Besides, the optimum load resistance at peak power density was lowered from 100 MΩ to 30 MΩ after the addition of carbon fillers due to the increased conductivity of the PVA-carbons composites after the addition of electrically conductive carbon fillers. Overall, it is observed that carbon fillers are effective in enhancing the tribopositive characteristic of PVA, and each carbon contributes to different magnitudes of enhancements. Next, we measured the dielectric and structural properties of PVA-carbons containing different carbon fillers and compared them to the TENG output metrics to identify the dominant factor contributing to the output enhancements.

**Fig. 2:**
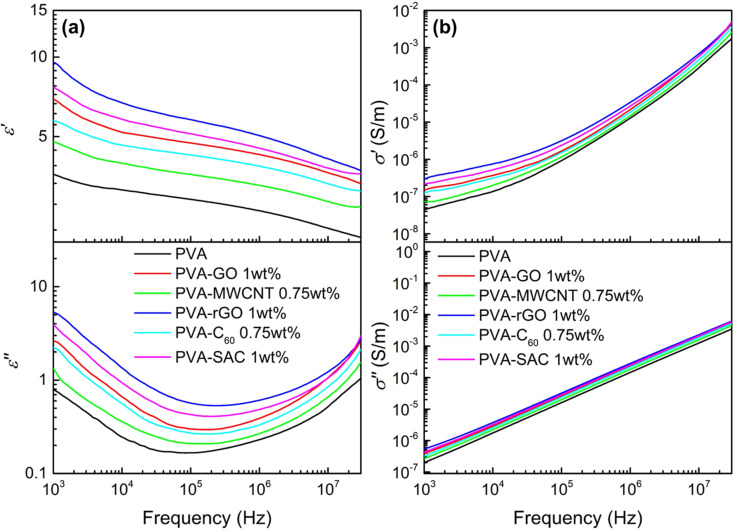
(a) Dielectric constant (*ε*′) and dielectric loss (*ε*′′), and (b) real (*σ*′) and imaginary (*σ*′′) electrical conductivity of pristine PVA and each PVA-carbon at the optimum loading concentration.

The incorporation of carbon fillers significantly altered PVA's electrical properties. The frequency-dependent dielectric behaviour, characterized by changes in dielectric constant (*ε*′) and dielectric loss (*ε*′′), is presented in Fig. 2(a) for PVA composites containing the optimal filler loading. It was reported that a higher dielectric constant enhances the ability of the material to accumulate surface charges during contact electrification, while a higher conductivity is usually attributed to enhancing the energy conversion or transfer efficiency by reducing the impedance.^35,36^Fig. 2(b) shows the frequency-dependent real and imaginary conductivity of the PVA and its composites. The complex conductivity, σ* of the material can be expressed as σ* = *σ*′ + i*σ*′′ where the real conductivity, *σ*′ = i*ωε*_0_*ε*′′ represents the in-phase component and the imaginary conductivity, *σ*′′ = i*ωε*_0_*ε*′′ represents the out-of-phase component with respect to the applied electric field. Here, *ω* = 2π*f*, and *ε*_0_ is the vacuum permittivity which is 8.854 × 10^−12^ Fm^−1^.^37^ The dielectric and conductivity spectra for all PVA-carbon composites at varying filler loadings are detailed in Fig. S3 and S4.† In contrary to our expectations, where the TENG enhancements are associated to high dielectric constants, the trends observed in these dielectric results do not correlate directly with the TENG output performance. Furthermore, the observed increases in dielectric constant and conductivity upon carbon filler addition were less pronounced than anticipated. This contrasts with our previous work employing CaCl_2_ as a filler, where enhanced electrical properties correlated with improved TENG performance.^38^ While the achieved electrical power output is comparable to our previous work employing CaCl_2_ as a filler, a significant discrepancy arises in dielectric properties. At 1 kHz, the dielectric constant (*ε*′) of PVA-rGO, for instance, is notably lower (*ε*′ = 9.5) compared to the previously reported value of 200 for PVA-CaCl_2_. Furthermore, a comprehensive literature review (see Table S1†) reveals that the dielectric constants of other polymer-carbon composites^39–44^ typically range from 10 to 105 at 1 kHz, exceeding the values observed in this study. These findings strongly suggest that dielectric properties alone cannot fully account for the observed enhancements in TENG performance, indicating the presence of other dominant factors.

**Fig. 3:**
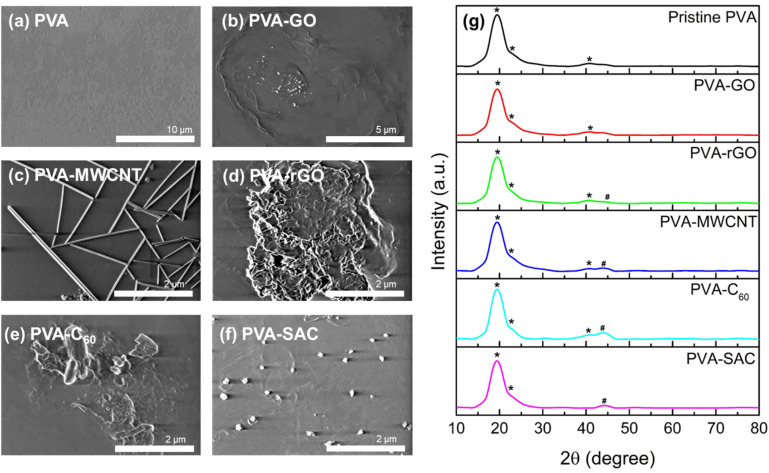
FESEM images of (a) pristine PVA, (b) PVA-GO, (c) PVA-MWCNT, (d) PVA-rGO, (e) PVA-C_60_, and (f) PVA-SAC composites. (g) The XRD spectra of pristine PVA and PVA-carbon composite at their optimal loading concentrations. Characteristic peaks of PVA are denoted with (*), while new peaks associated with the respective carbon fillers are marked with (#).

To elucidate the underlying cause of the unexpected dielectric behaviour, a comprehensive structural analysis was undertaken using field emission scanning electron microscopy (FESEM). The morphology of the various carbon fillers within the PVA matrix is depicted in Fig. 3(a–f). MWCNT and SAC show good dispersion within the matrix, whereby their respective individual tube-like structures and particle-like structures can be observed. On the other hand, slight agglomeration is observed for C_60_, whereas a higher degree of stacking and agglomeration of sheet-like structures are observed for both rGO and GO. However, the various degrees of dispersion do not show an obvious trend which can be related to the electrical outputs. In particular, MWCNT, which is highly dispersed within the PVA matrix, generated the second lowest electrical and power outputs. In contrast, rGO, which experienced stacking and agglomeration, has superior electrical outputs compared to MWCNT. Aside from visualizing the degree of filler dispersion, XRD was also carried out to determine the changes in crystallinity after the incorporation of carbon fillers. The XRD spectra of the pristine PVA and PVA-carbons are depicted in Fig. 3(g). Characteristic peaks of PVA, which are denoted with (*), are observed for all spectra, including a strong broad peak at 19.35° representing the (101) plane, a shoulder peak at 22.65° representing the (200) plane, and a weak at 40.85° representing the (111) plane.^45^ For PVA-rGO, PVA-MWCNT, PVA-C_60_ and PVA-SAC, new peaks which are associated to the respective carbon fillers are observed, which are denoted by #.^46–48^ The XRD patterns exhibited consistent, broad peaks corresponding to the (101) and (200) planes across all samples, indicating no significant changes in crystallinity upon carbon filler incorporation. Consequently, neither filler dispersion nor crystallinity can be attributed as the primary factors influencing the TENG performance enhancements.

**Fig. 4:**
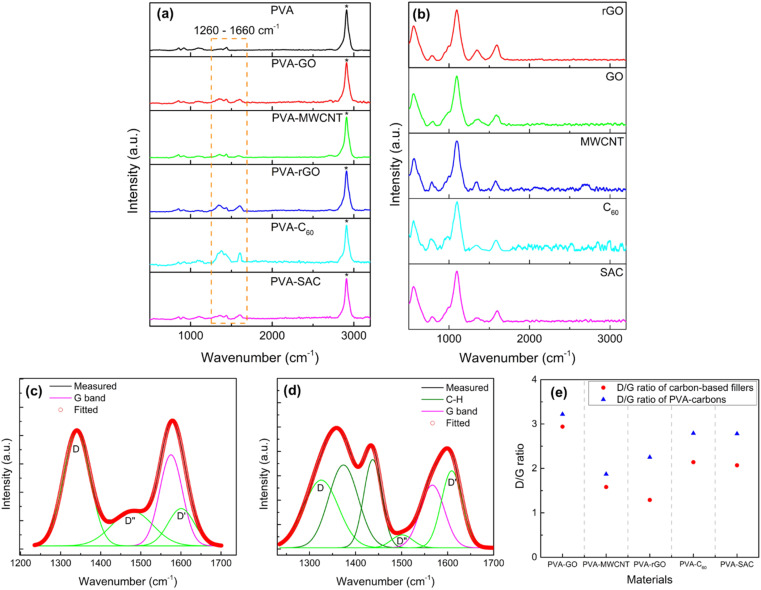
Raman spectra of carbon fillers (a) and PVA-carbon composites at optimal loading concentration (b). Deconvolution of D and G bands for super activated carbon (SAC) filler (c) and its corresponding PVA-SAC composite (d). Summary of the D/G ration for all carbon fillers and their PVA-carbon composites at optimal filler wt% (e).

To elucidate the nature of interactions between the carbon fillers and the PVA matrix, Raman and FTIR spectroscopy were employed. Raman spectra of the pristine carbon fillers and PVA-carbon composites, exhibiting characteristic G, D, D′ and D′′ bands at 1583 cm^−1^, 1350 cm^−1^, 1620 cm^−1^, and 1480 cm^−1^ respectively, are presented in Fig. 4(a) and (b). The G band of carbon fillers represents the in-plane vibration of the sp^2^ hybridized carbon atoms arranged in hexagonal lattices like graphene, and it is electrically conductive due to the π electrons roaming freely within the plane of the materials.^49^ Meanwhile, D, D′ and D′′ bands of carbon fillers are characteristic to the structural defects such as vacancies or edges in the hexagonal lattice.^50^ These defects are usually of sp^3^ hybridization, which disrupt the electrical conductivity by restricting the π electrons movements. Thus, the analysis of the D, D′, D′′ and G bands is highly relevant for us to unravel the anomalies in the dielectric and conductivity properties of the PVA-carbons. We deconvoluted the broad peak at around 1600 cm^−1^ to separate the D and G bands into individual peaks. The relative amounts of defective (sp^3^) and conductive (sp^2^) structures are quantified by dividing the areas under the peaks of D, D′ and D′′ bands by the G band peak and expressing them as D/G ratios. An example of the deconvolution is shown in Fig. 4(c) (for SAC filler) and (d) (for the corresponding PVA-SAC composite), while the remaining deconvoluted graphs are compiled in Fig. S5 and S6 (see the ESI†). A higher D/G ratio indicates a lower conductivity due to the presence of more defective structures, while a lower D/G ratio indicates a higher conductivity due to the presence of more conductive structures. From Fig. 4(e), it is observed that the D/G ratios of the PVA-carbons are higher than the respective carbon fillers, indicating that the carbon fillers interact with the PVA host matrix by saturating the conductive sp^2^ structures into defective sp^3^ structures. This reduces the amount of free-roaming π electrons from the sp^2^ hybridized carbon atoms, lowering the conductivity of the carbon fillers. Thus, the increment in the dielectric and conductive properties of PVA after the incorporation of carbon is lower than expected as there are less π electrons available to boost the charge responsiveness and conductivity of the composite. Thus, by corroborating the dielectric and the Raman measurements, we infer that the enhancements in electrical outputs arise from the interactions between the carbon fillers and the PVA host polymer.

**Fig. 5:**
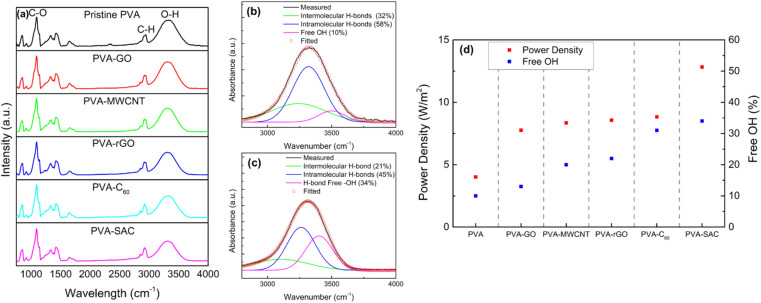
(a) Fourier Transform Infrared (FTIR) spectra of pristine PVA and PVA-carbon composites. (b) and (c) Deconvolution of the FTIR spectra for pristine PVA and PVA-SAC, respectively. (d) Correlation between power density and the percentage of free –OH groups in pristine PVA and PVA-carbon composites at their optimal filler weight percentages.

Since the structural interactions between PVA and the carbon fillers are essential in enhancing the triboelectric outputs, Fourier transform infra-red (FTIR) spectroscopy was conducted to further understand the nature of these interactions. The FTIR spectra ranging from 650 cm^−1^ to 4000 cm^−1^ are shown in Fig. 5(a). Sharp peaks are observed at the wavenumbers of 1090 cm^−1^ and 2942 cm^−1^, which are attributed to the C–O and C–H stretching vibration respectively, while the broad peak at 3000 to 3700 cm^−1^ represents the O–H stretching vibration.^51–53^ Comparison between pristine PVA and PVA-carbon composites shows differences in the intensities of the O–H stretching vibration peak, indicating that the sp^3^ hybridization of the carbon fillers is due to their reaction with the –OH functional groups in PVA. The O–H vibration broad peak is a combination of three peaks, which are attributed to the intermolecular and intramolecular hydrogen bonding and free hydroxyl (–OH) groups, located at 3250 cm^−1^, 3450 cm^−1^, and 3550 cm^−1^ respectively.^54^ Therefore, to quantify the changes in the degree of hydrogen bonding due to the interactions between the carbon and PVA, the FTIR spectra were converted to absorbance using Beer Lambert's Law (as discussed in the Experimental section), and the broad O–H vibration peak was deconvoluted into the three smaller peaks. The exemplary convoluted FTIR spectra of pristine PVA and PVA-SAC are shown in Fig. 5(b) and (c), while the other PVA-carbons FTIR spectra are shown in Fig. S7.† The percentage of each –OH group was calculated by the ratio of the area under each peak and the total area under all three peaks. The comparison of the degree of hydrogen bonding and free –OH groups across the PVA-carbons is shown in Fig. 5(d). Incorporating carbon fillers into PVA disrupts the hydrogen bonding network, resulting in a reduction in the hydrogen bonded –OH groups and increasing the amount of free hydroxyl group. This reduction stems from the interaction of sp^2^ carbons with the oxygen atom, lowering its electronegativity and hindering its ability to form H-bonds with other –OH groups. Notably, pristine PVA exhibits the lowest percentage of free –OH groups (10%), followed by PVA-GO (13%), PVA-MWCNT (20%), PVA-rGO (22%), PVA-C_60_ (31%), and PVA-SAC (34%). This trend is parallel to the observed electrical and power outputs of the TENGs, suggesting that the high quantity of free –OH functional groups play a crucial role in enhancing the tribopositive characteristic of the PVA-carbons.

To further understand how temperature affects the hydrogen bonding network and the number of free –OH groups, we performed temperature-dependent FTIR measurements on both pristine PVA and PVA-SAC samples. After careful baseline correction and subtraction of the air reference, the results were obtained and are presented in Fig. S8.† Several key observations can be drawn from Fig. S8.† As the temperature rises, the intensity of the C–OH peak at ∼1650 cm^−1^ diminishes for both samples, signifying a weakening of hydrogen bonds involving the hydroxyl group.^55^ Concurrently, the O–H peak around 3345 cm^−1^ shifts to higher wavenumbers (3372 cm^−1^) and decreases in intensity, indicating a reduction in hydrogen bond strength and an increase in the number of free –OH groups. To delve deeper into the hydrogen bonding dynamics, we deconvoluted the broad peak centered around 3300 cm^−1^ to quantify the total area under the curve at various temperatures. Fig. S10† illustrates these results. Subsequently, we normalized the total area of the –OH peak at each temperature to the reference value at 25 °C (*A*/*A*_0_). As depicted in Fig. S10,† PVA-SAC exhibits a more pronounced decrease in normalized area with increasing temperature compared to pristine PVA. This observation suggests that the incorporation of SAC into the PVA matrix significantly disrupts the hydrogen bonding network, and this effect becomes more pronounced at elevated temperatures. These findings are consistent with our hypothesis that disrupting the hydrogen bonding network with carbon fillers leads to an increase in the number of free –OH groups, which is crucial for enhancing the triboelectric performance of PVA-based composites. The temperature-dependent study further emphasizes the dynamic nature of hydrogen bonding and its impact on the material's properties. Notably, the more pronounced changes observed in PVA-SAC samples suggest that the presence of carbon fillers accelerates the disruption of the hydrogen bonding network at elevated temperatures.

**Fig. 6:**
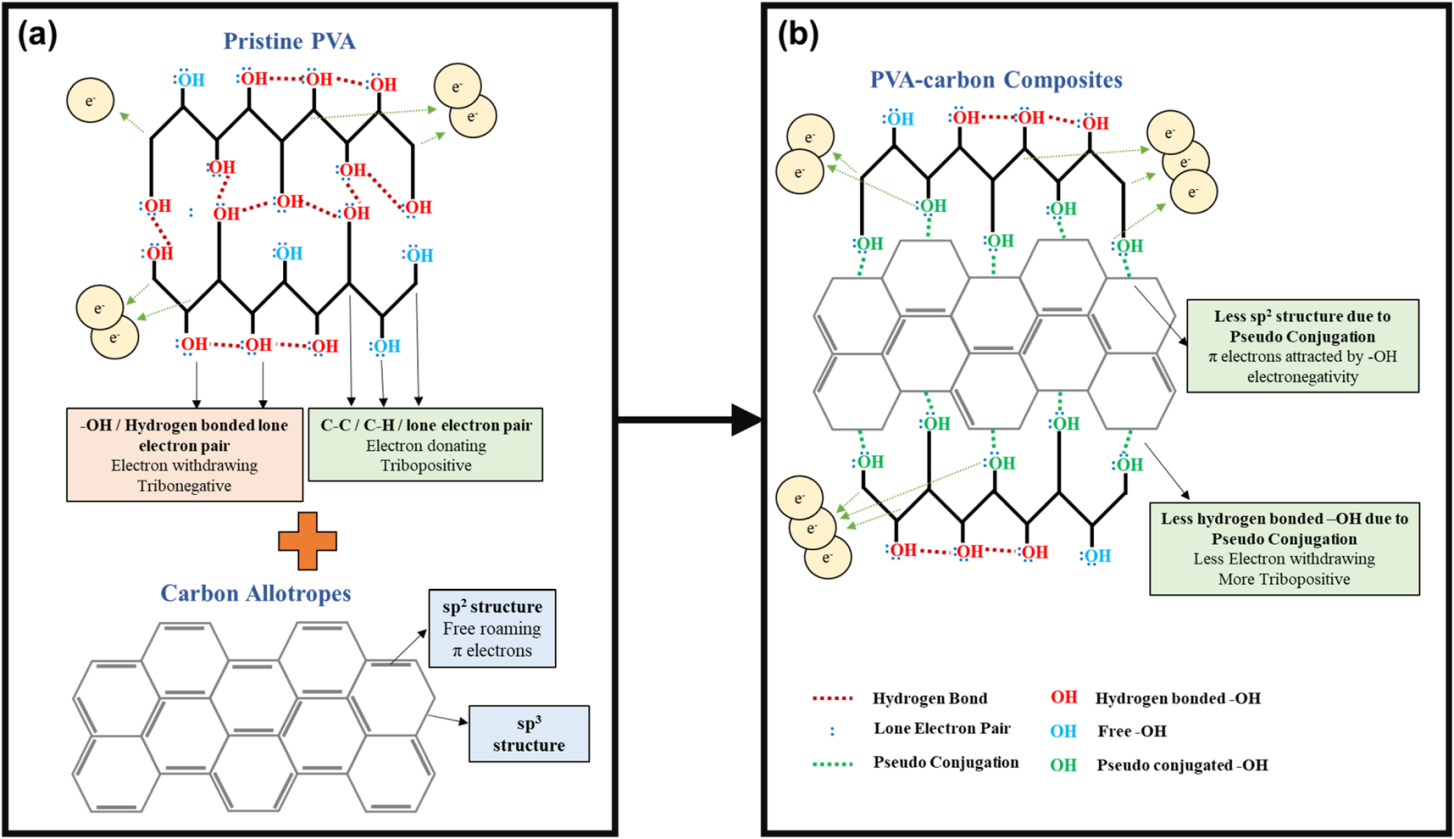
Proposed bonding mechanism of (a) pristine PVA and (b) PVA-carbon composites, illustrating the potential interactions that increase the availability of free –OH groups in the composite materials.

A deeper understanding of this interaction mechanism is essential to further optimize device efficiency. The illustration in Fig. 6 compares the conditions of the –OH functional groups in pristine PVA with those in the PVA-carbons. In pristine PVA, the oxygen atom is fully saturated with two pairs of lone electrons, and the –OH functional groups mostly interact with each other through inter- or intramolecular hydrogen bonding as shown in Fig. 6(a). The –OH groups are highly electron-withdrawing, thus hindering the transfer of electrons from PVA towards SR during the contact electrification (CE) process. In this case, the main electron donors are from the fully saturated C–H and C–C bonds as they have higher electron donating tendencies compared to the electronegative –OH group. On the contrary, the –OH functional groups in PVA-carbons have a lower degree of hydrogen bonding. This reduction stems from the interaction between the carbon fillers and the –OH functional groups, which occupies the electrons from the oxygen atom and prevents it from forming hydrogen bonds between adjacent –OH functional groups. Corroborating with the Raman results, we theorize that the free-roaming π electrons from the sp^2^ structure form weak interactions with the lone electron pairs on the oxygen atoms. This prevents the π electrons on the carbon fillers from roaming freely and transforming them into sp^3^-like defective structures. The observed interactions between carbon fillers and PVA oxygen atoms resemble a weakened form of conjugation, as the oxygen atoms are not covalently linked to the sp^2^ carbon network within the fillers (see Fig. 6(b)). We term these interactions as “pseudo conjugations”. Within the composites, the ability of different fillers to engage in pseudo conjugation with the polymer matrix varies based on the availability of sp^2^ electrons on their surfaces. MWCNTs, with much less –OH functional groups participate in a lower extent of hydrogen bonding compared to graphene oxide, steadily increasing the degree of pseudo conjugation. Graphene oxide, rich in COOH and –OH groups, readily forms hydrogen bonds with PVA molecules, which results in a lower degree of pseudo conjugation. This trend continues, with rGO, C_60_, and SAC showing successively less capacity for hydrogen bonding due to a diminishing number of COOH and –OH functional groups while having a higher capacity of pseudo conjugation.

**Fig. 7:**
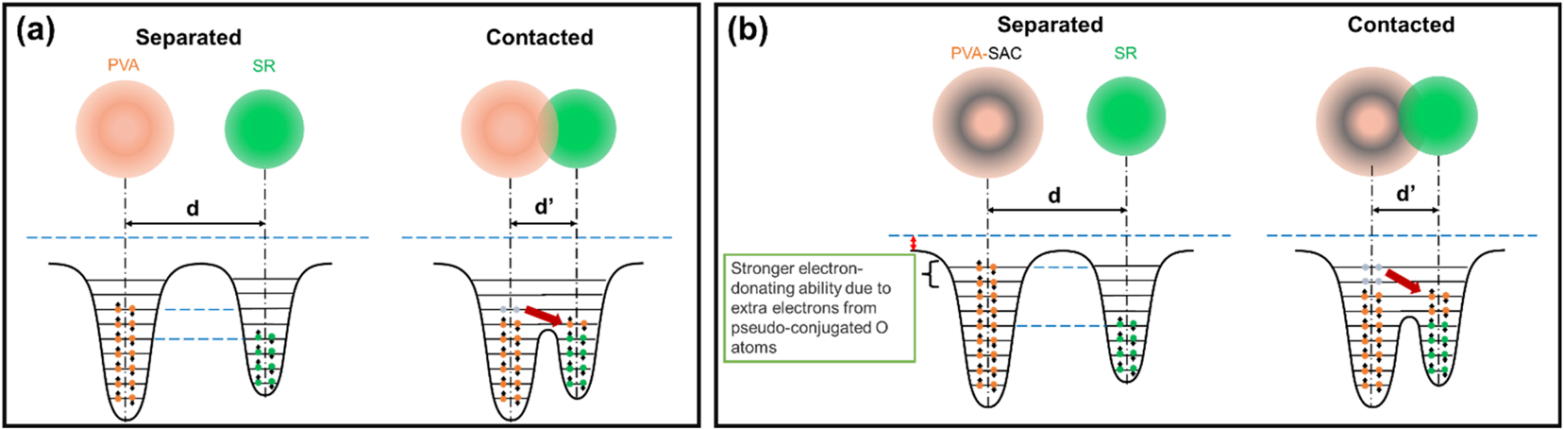
OEC model describing the electron transfer during contact electrification between silicone rubber and (a) pristine PVA, and (b) PVA-SAC, respectively.

The pseudo conjugation between the oxygen atoms and the sp^2^ carbon atoms will hinder the electron-withdrawing effect of the oxygen atoms while improving their electron-donating ability at the same time. Oxygen atoms with stronger electron-donating abilities can participate in the CE process by donating their lone electron pairs to the SR, contributing to the overall electron-donating ability of the PVA composites. This enhancement of electron-donating ability is illustrated using overlapped electron cloud (OEC) models.^55,56^ The OEC models of pristine PVA and PVA-SAC, which are shown in Fig. 7(a) and (b), serve as illustrative examples throughout this explanation, representing the extrema of PVA composites investigated. As depicted in Fig. 7(a), the electron orbitals of PVA and SR are shown to overlap during the contact of both materials, fusing the two distinct potential wells into an asymmetric double-well. In this state, the potential barrier between the two molecules is diminished. The higher electron energy levels in PVA provide an energy gradient, driving the electrons to flow towards SR, resulting in the generation of positive surface charges in PVA and negative surface charges in SR. After the addition of carbon fillers and the occurrence of pseudo conjugations, the overall tribopositivity of PVA-SAC is enhanced compared to pristine PVA. More electrons from the pseudo-conjugated oxygen atoms are available to participate in CE, which is depicted by the extra pair of electrons in the PVA-SAC potential well (Fig. 7(b)). Consequently, during the overlap of the electron clouds, more electrons are driven from PVA-SAC to SR, increasing the surface charge density generated. In short, based on the structural analyses from the Raman and FTIR measurements, we theorize that the pseudo conjugation between the carbon fillers and PVA improves its electron-donating ability, which ultimately enhances the electron transfer during CE as elucidated by the OEC model.

**Fig. 8:**
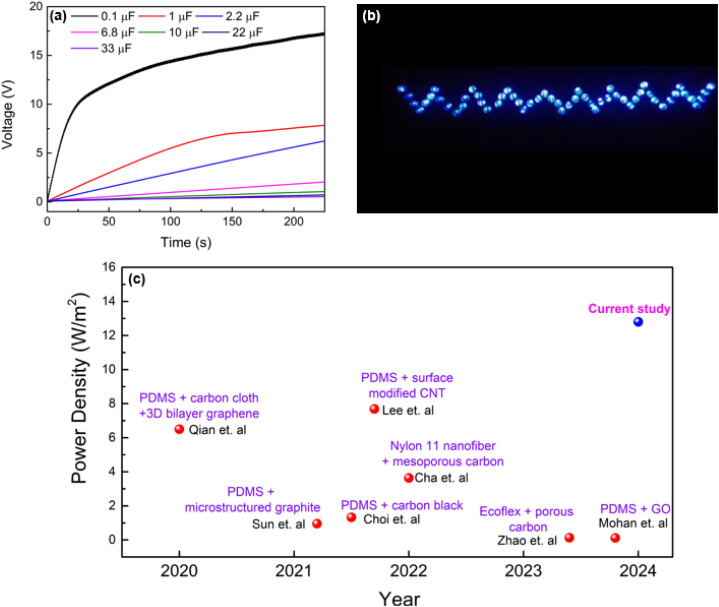
Device performance. (a) Capacitor charging curve of the PVA-SAC/SR TENG. (b) PVA-SAC/SR TENG powering LEDs during contact-separation cycles. (c) Comparison of power densities with previous studies.

The energy harvesting capabilities of the PVA-carbon composite TENGs were evaluated through capacitor charging and LED lighting demonstrations. Capacitors ranging from 0.1 μF to 33 μF were charged using the PVA-SAC/SR TENG under a contact-separation motion of 1 Hz with a force of 50 N. The TENG demonstrates its ability to quickly charge a 0.1 μF capacitor to over 10 V within 25 seconds (25 cycles), as shown in Fig. 8(a). This rapid charging highlights the device's potential for applications requiring short-term energy storage. Although larger capacitors (1 μF) exhibited slower charging times, reaching 1.5 V in 25 seconds and 6.5 V in 125 seconds, the TENG demonstrated efficient charging capabilities for smaller capacitors commonly employed in low-power IoT devices. This highlights the TENG's potential as a suitable power source for such applications. Fig. 8(b) showcases 55 commercial LEDs illuminated by the PVA-SAC/SR TENG. ESI Video 1† demonstrates the TENG's ability to power these LEDs at frequencies ranging from 1 to 4 Hz (with a 50 N contact force), highlighting its potential for powering miniature IoT devices. To further investigate the relationship between contact force and light intensity, we varied the contact force from 20 N to 50 N (ESI Video 2†). Visually, we observed that increasing the contact force led to a higher light intensity (Fig. S11†). In contrast, varying the contact frequency from 1 Hz to 4 Hz had minimal impact on the light intensity, as demonstrated in ESI Video 1†. Fig. 8(c) showcases the advancement in TENG power density where it compares our best-performing TENG (PVA-SAC/SR) with devices reported in recent literature utilizing carbon fillers as fillers. It highlights a clear trend of increasing power density across various carbon filler-based TENGs from 2020 to 2024. Our TENG significantly outperforms previous studies, highlighting the crucial role of selecting optimal carbon fillers in the tribopositive contact layer for maximizing power generation.

## 4 Conclusions

In summary, this study explores the ideal carbon filler to unlock superior performance in PVA-based TENGs. We compared five different PVA/carbon fillers composites and their contributions towards enhancing the electrical outputs of TENGs in an increasing order: GO < MWCNT < rGO < C_60_ < SAC. This investigation successfully isolates the key factors influencing electrical outputs by eliminating the influence of the dielectric constant, degree of filler dispersion, and degree of crystallinity. Raman spectral analysis indicated a notable increase in the D/G ratio for PVA-carbon composites compared to pristine carbon fillers, indicating a transformation from conductive sp^2^ to less conductive sp^3^ hybridized carbon within the composite structure. FTIR analysis unveiled a correlation between enhanced TENG performance and an increased population of free hydroxyl (–OH) groups within the PVA-carbon composites. This suggests that the interaction between carbon fillers and PVA, potentially involving the formation of sp^3^ hybridized carbon structures through pseudo-conjugation with PVA's –OH groups, plays a pivotal role in enhancing electron donation and, consequently, TENG output. The disruption of hydrogen bonding within the PVA matrix, evidenced by an increased D/G ratio and a reduction in hydrogen-bonded –OH groups, is attributed to the interaction between carbon fillers and PVA. This modification enhances the concentration of free –OH groups, thereby increasing the material's electron-donating capacity and tribopositive nature. Consequently, the PVA-SAC/SR TENG exhibited a remarkable 220% increase in power density (12.8 W m^−2^) compared to the pristine PVA/SR TENG. These findings underscore the pivotal role of filler-matrix interactions in optimizing TENG performance and offer new avenues for developing advanced triboelectric materials.

## Data availability

The data used in this study are available from the corresponding author, T. S. Velayutham, upon reasonable request.

## Author contributions

Jian Ye Cheong: methodology, formal analysis, investigation, visualization, writing – original draft. Jason Soon Chye Koay: methodology, formal analysis, investigation, visualization, writing – original draft. Sanjeev Raja Gopal: methodology, formal analysis, investigation. Thamil Selvi Velayutham: conceptualization, methodology, resources, formal analysis, investigation, writing – review & editing, supervision, project management. Wee Chen Gan: conceptualization, methodology, resources, formal analysis, investigation, writing – review & editing, supervision, project management.

## Conflicts of interest

There are no conflicts to declare.

## Supplementary Material

NA-007-D4NA00820K-s001

NA-007-D4NA00820K-s002

NA-007-D4NA00820K-s003
