# Supplementary material for: Enhancing the tribopositive characteristics of polyvinyl alcohol (PVA)-carbon composites by optimizing the PVA-carbon interaction with various carbon fillers
Source: Nanoscale Adv. 2024 Dec 9;7(3):819–29. doi: 10.1039/d4na00820k (PMC11647576; doi:10.1039/d4na00820k)
Supplement: NA-007-D4NA00820K-s001 [file NA-007-D4NA00820K-s001.pdf]

Supplementary Information (SI) for Nanoscale Advances.
This journal is © The Royal Society of Chemistry 2024

Supplementary Information

Enhancing the tribopositive characteristics of polyvinyl alcohol (PVA)-carbon composites by optimizing the PVA-carbon interaction with various carbon fillers

Jian Ye Cheong [a], Jason Soon Chye Koay [b, c], Sanjeev Raja Gopal [a], Thamil Selvi Velayutham [a, *], Wee Chen Gan [b, c, *]

[a] *Low Dimensional Materials Research Center, Department of Physics, Faculty of Science, Universiti Malaya, 50603 Kuala Lumpur, Malaysia*

[b] *School of Energy and Chemical Engineering, Xiamen University Malaysia, Selangor Darul Ehsan 43900, Malaysia*

[c] *Kelip-kelip! Center of Excellence for Light Enabling Technologies*

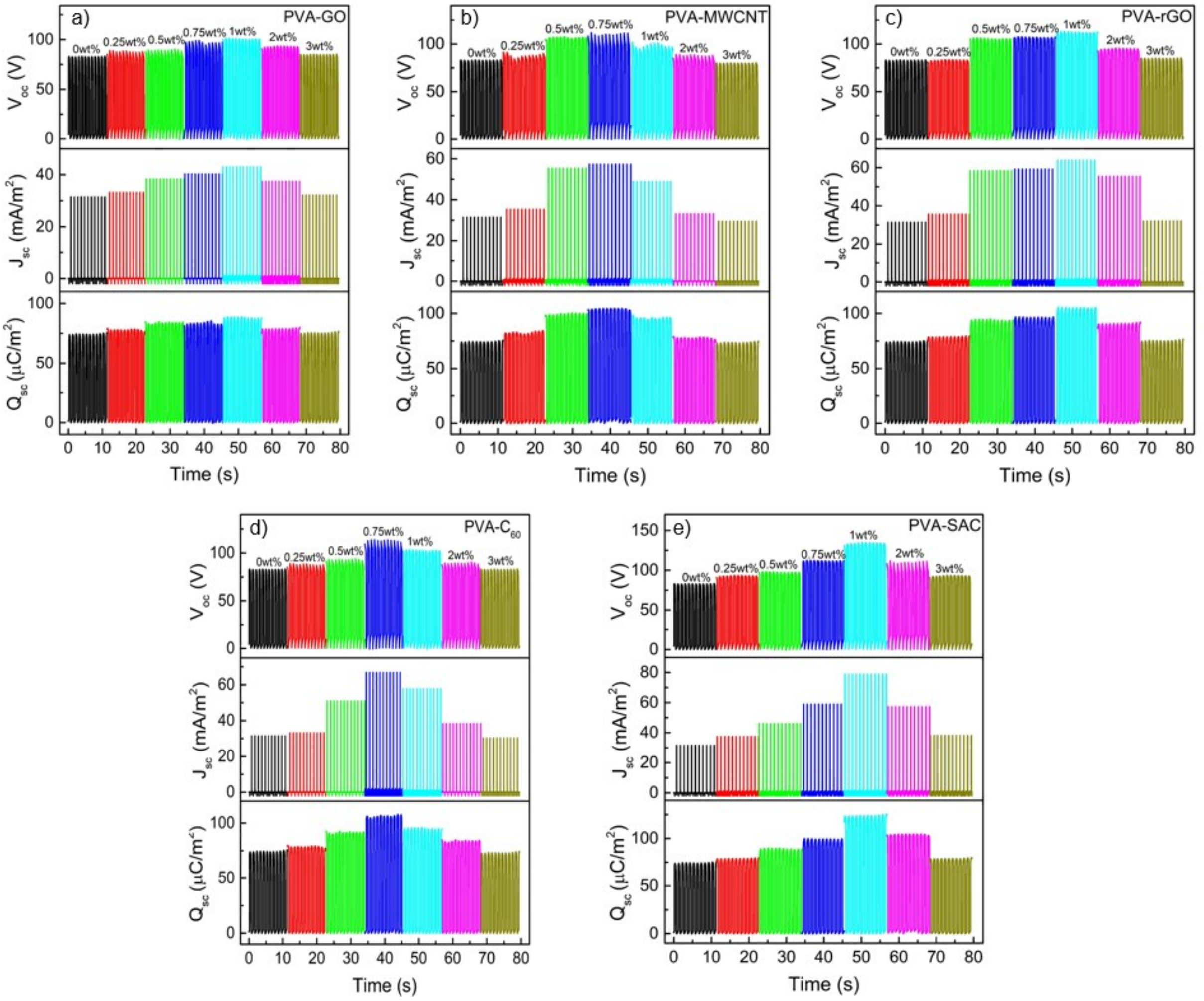

Figure S1: Open circuit voltage (V_{oc}), short-circuit current density (J_{sc}) and short-circuit charge density (Q_{sc}) of pristine PVA and each PVA-carbon at loading concentrations from 0.25 wt% to 3 wt%.

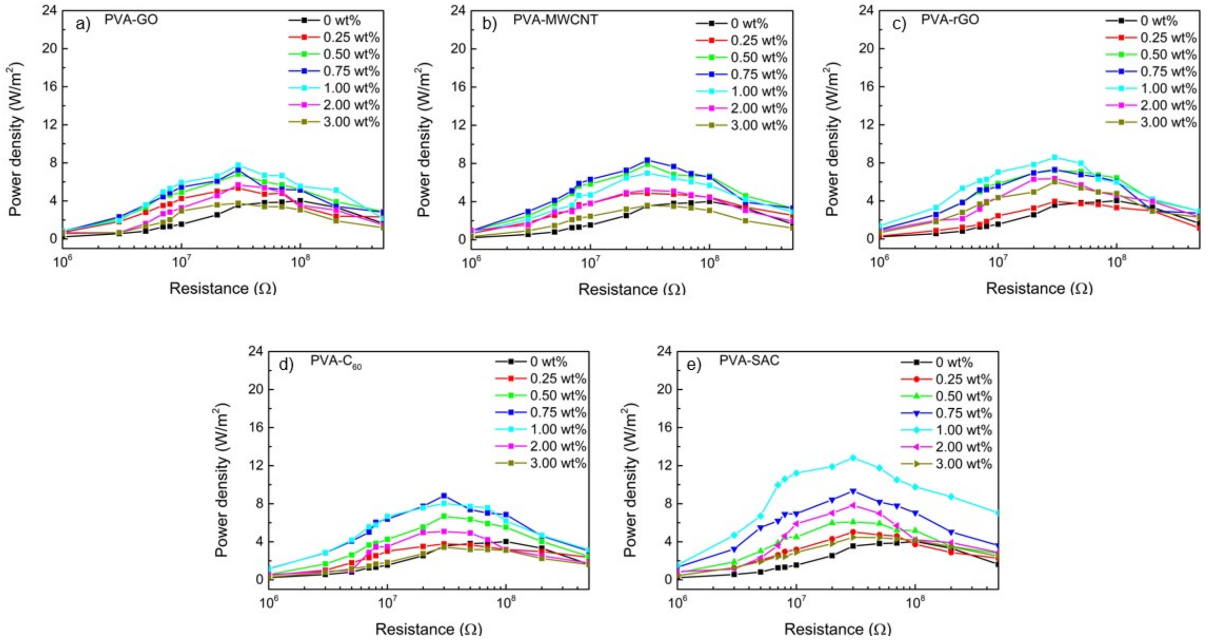

Figure S2: Power density curves of pristine PVA and each PVA-carbon at loading concentrations from 0.25 wt% to 3 wt%.

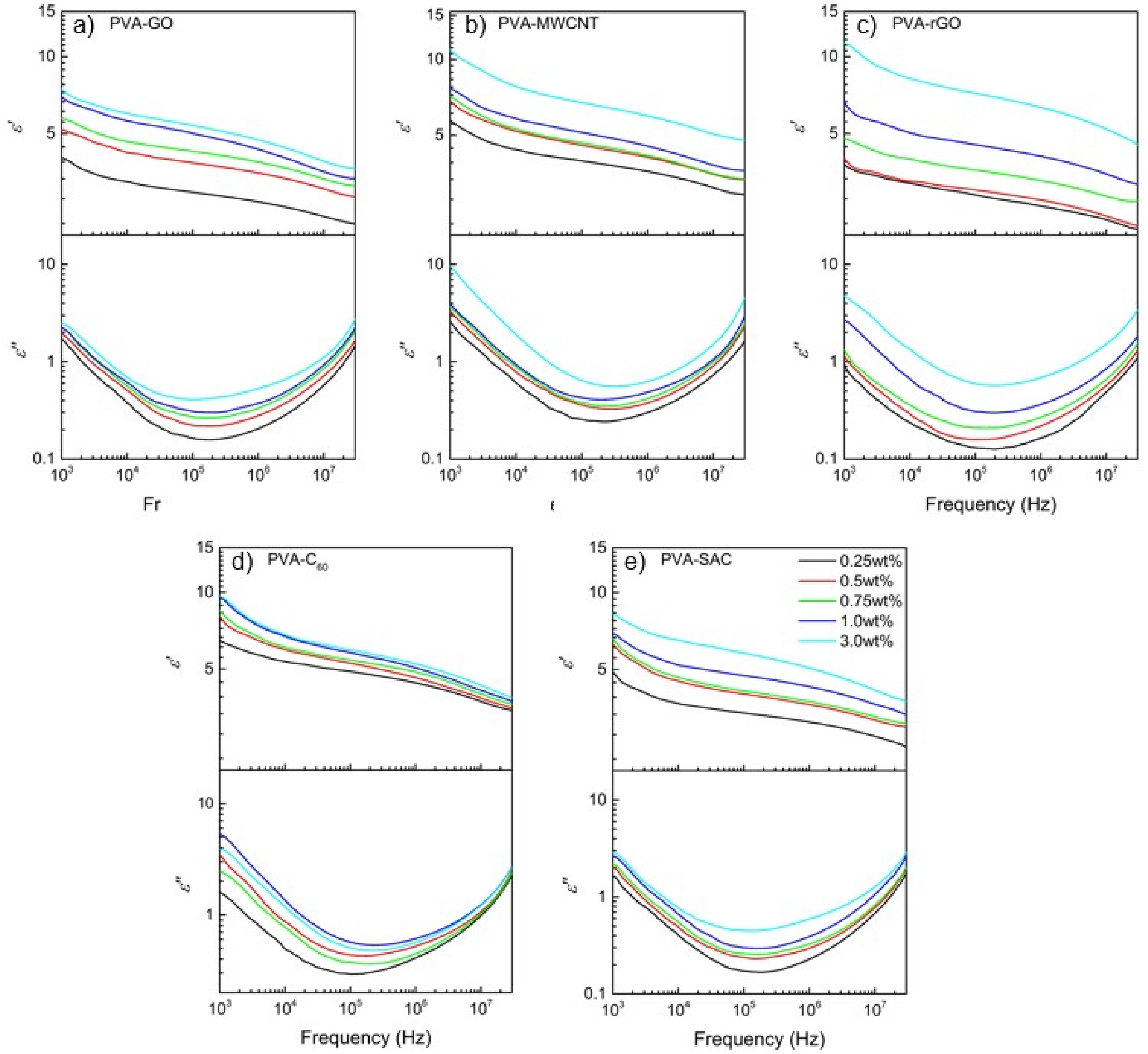

Figure S3: Dielectric constant and dielectric loss of pristine PVA and each PVA-carbon at loading concentrations from 0.25 wt% to 3 wt%.

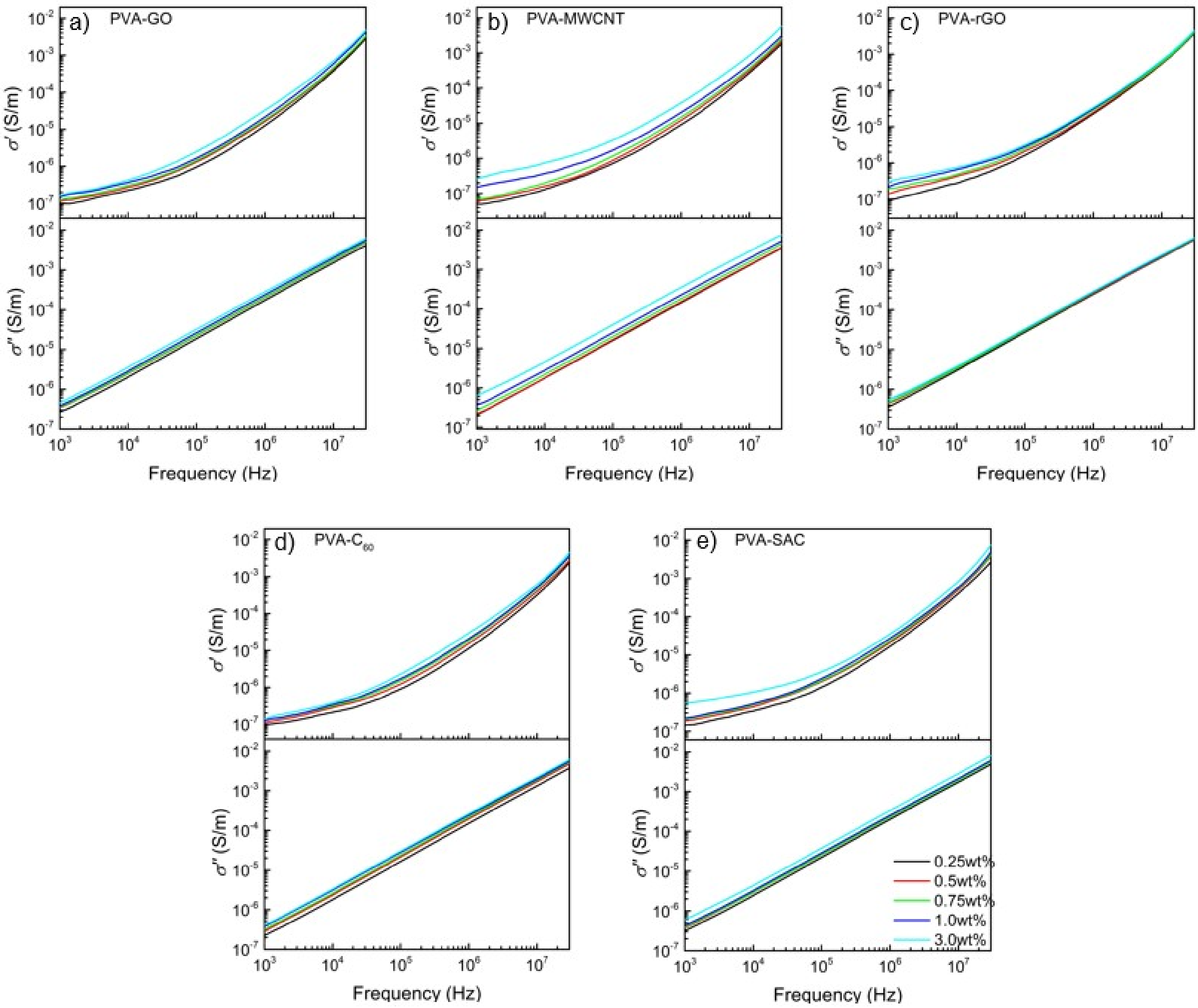

Figure S4: Real and imaginary conductivity of pristine PVA and each PVA-carbon at loading concentrations from 0.25 wt% to 3 wt%.

Table S1: Typical dielectric constants at 1kHz of polymers before and after the addition of carbon-based fillers.

Host polymer	Carbon filler	Loading concentration	Host polymer's dielectric constant	Composite's dielectric constant	References
PVA	rGO	1.00 wt%	3.6	9.6	Current Work
PVA	SAC	1.00 wt%		7.7	
PVA	GO	1.00 wt%		6.9	
PVA	C_{60}	0.75 wt%		5.8	
PVA	MWCNT	0.75 wt%		4.8	
Ethylene vinyl acetate copolymer	CNT	1.00 vol%	<10	>450	Mittal et al. 2023 [1]
Polyurethane /polyaniline (70/30) copolymer	rGO	2.00 wt%	100	>800	Dash et al. 2023 [2]
PDMS	MWCNT	1.00 wt%	2.6	>400	Liu et al. 2020 [3]
Poly-propylene	MWCNT	2.56 vol%	2.4	>3000	Ameli et al. 2014 [4]

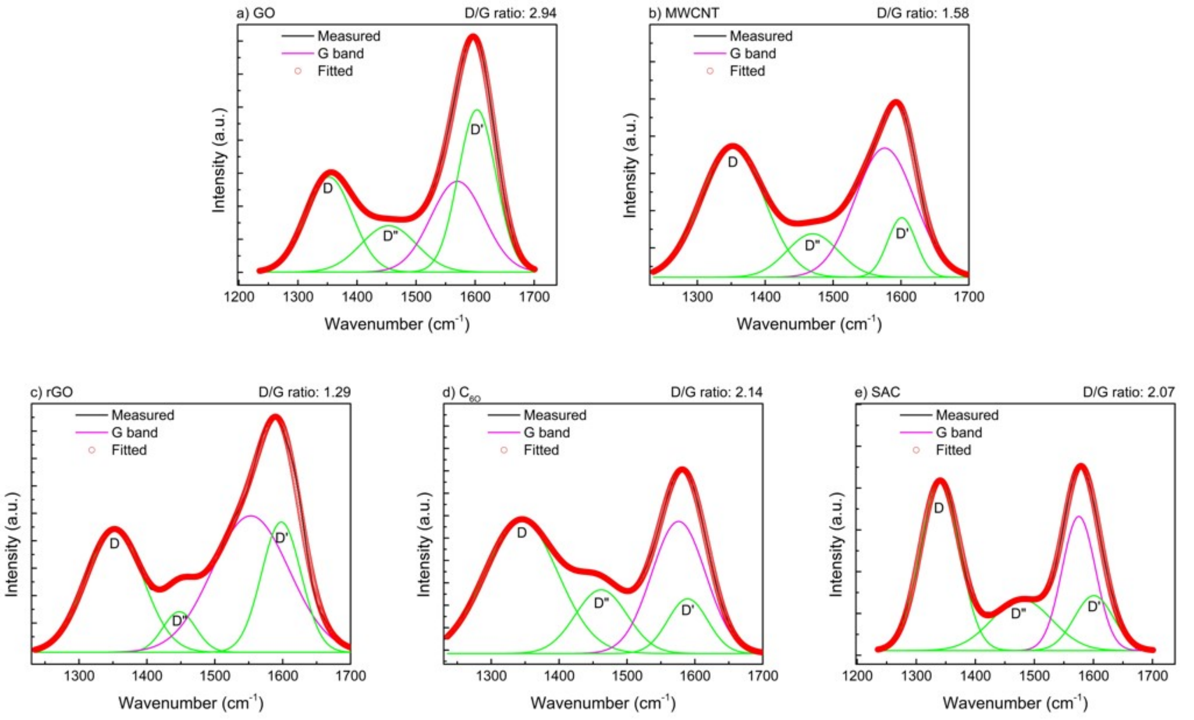

Figure S5: Peak deconvolution of the Raman spectra and calculated D/G ratio of each carbon allotrope.

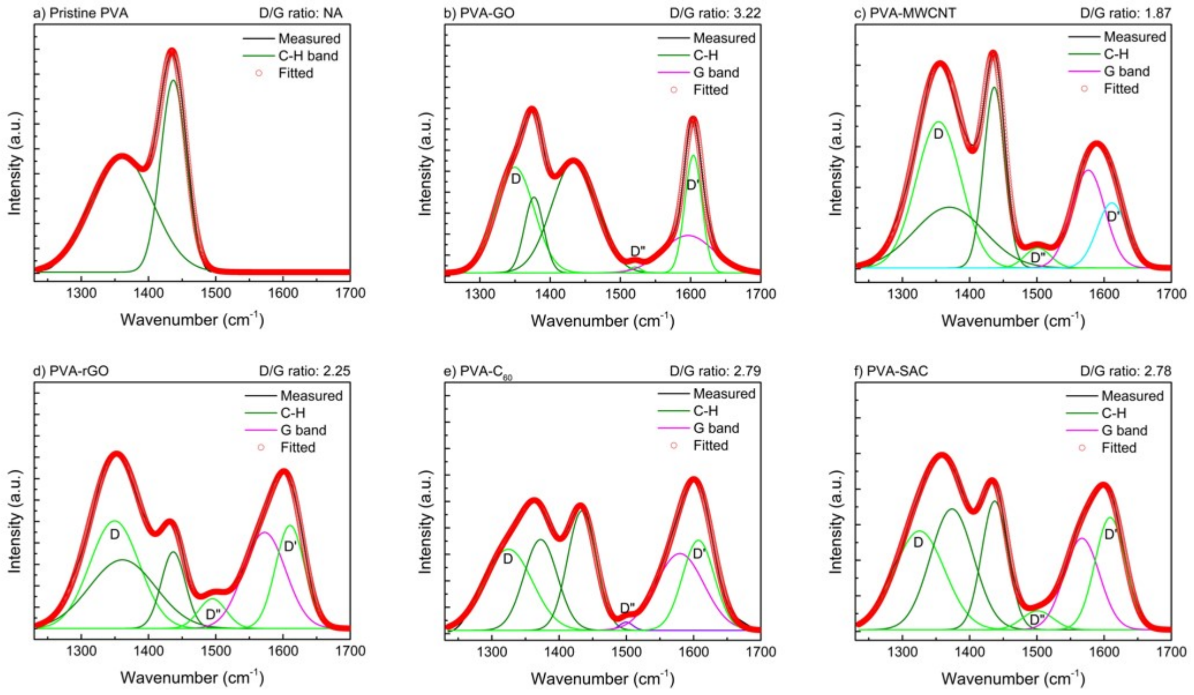

Figure S6: Peak deconvolution of the Raman spectra and calculated D/G ratio of each PVA-carbon.

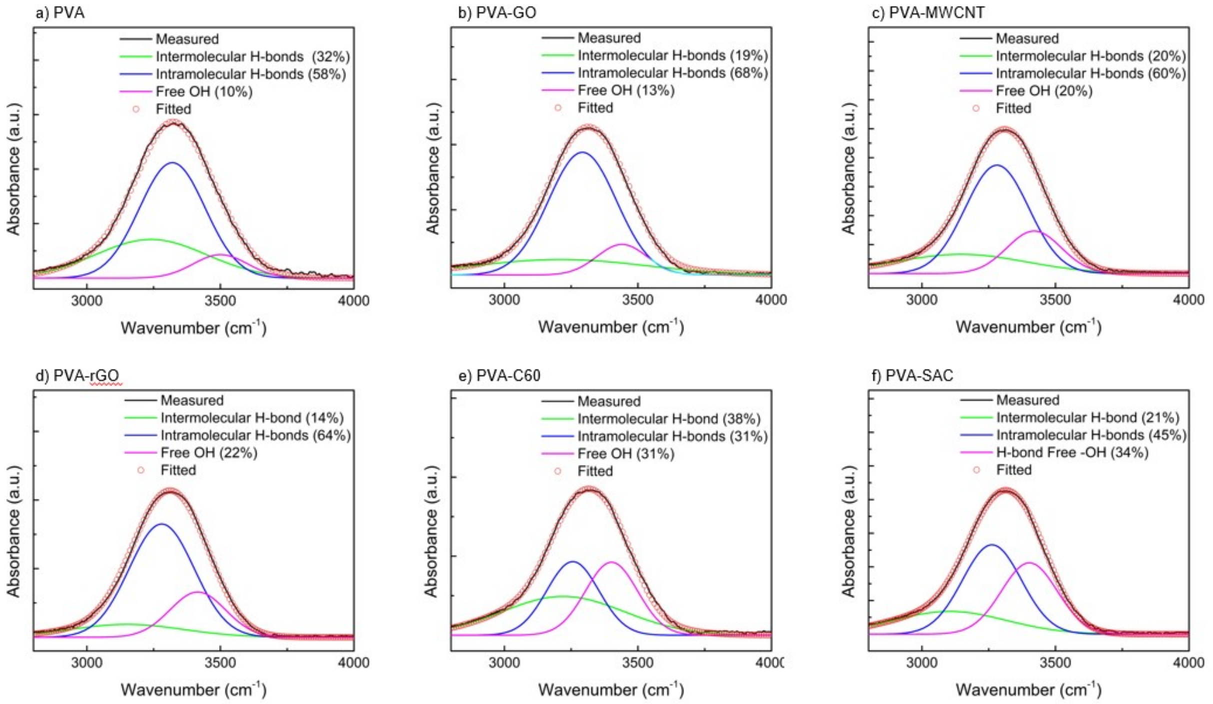

Figure S7: Peak deconvoluted FTIR spectra of pristine PVA and each PVA-carbon.

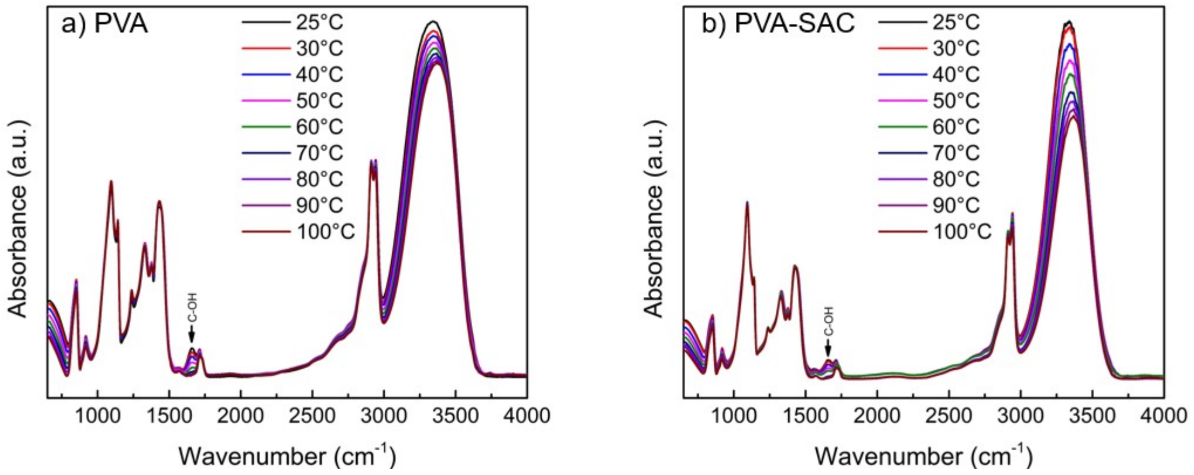

Figure S8: Temperature dependent FTIR for pristine PVA and PVA-SAC ranging from 25°C to 100°C.

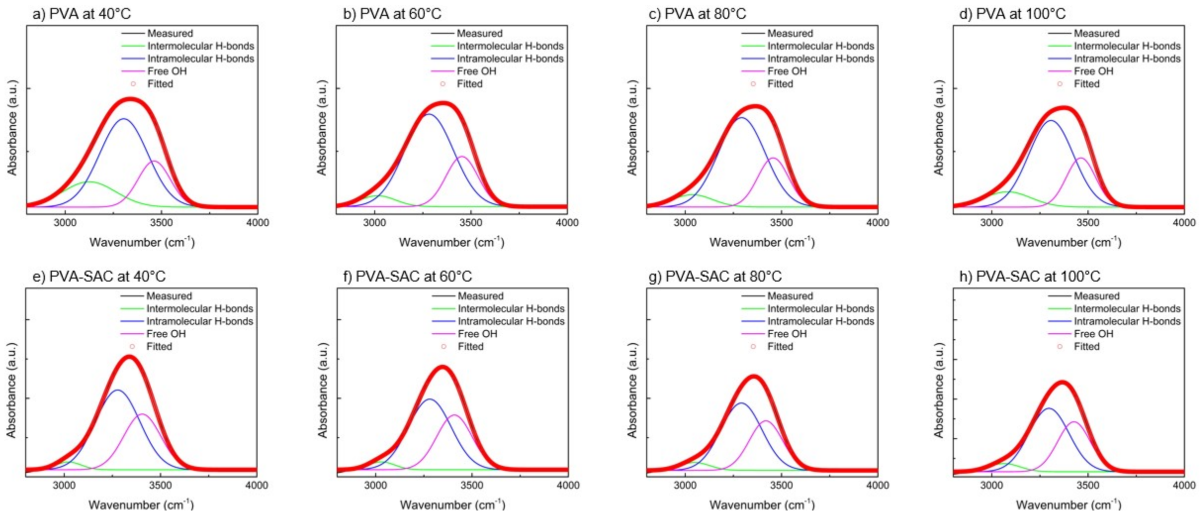

Figure S9: Deconvolution of temperature-dependent FTIR spectra of pristine PVA and PVA-SAC at 40°C, 60°C, 80°C and 100°C.

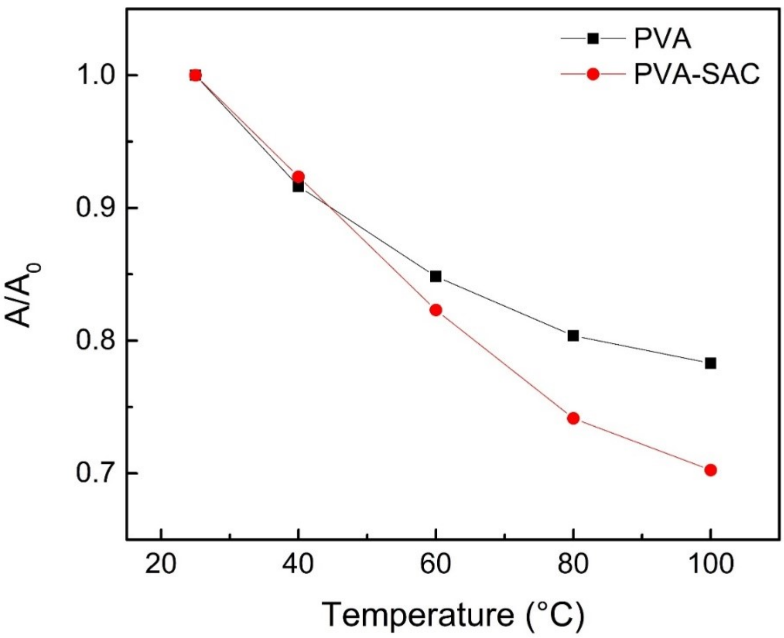

Figure S10: Normalized area under the curve (A/A_0) as a function of temperature for pristine PVA and PVA-SAC. A represents the area under the curve at 25°C, while A_0 denotes the area under the curve at the specified temperature.

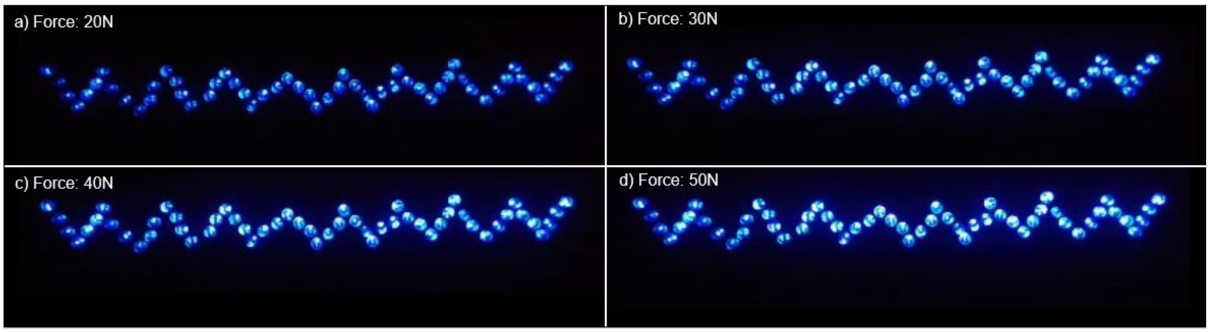

Figure S11: PVA-SAC/SR TENG lighting up LEDs during CS under different forces.

Table S2: SEM images of pristine PVA at 5k, as well as PVA-GO, PVA-MWCNT, PVA-rGO, PVA-C60, and PVA-SAC at magnification of 10k, 20k and 50k.

**Sample**	**FESEM Images**		
**Pristine PVA**	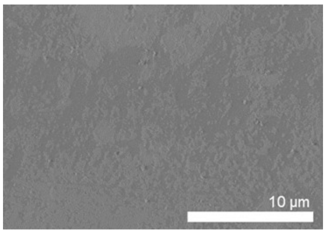 5k magnification		
**PVA-GO**	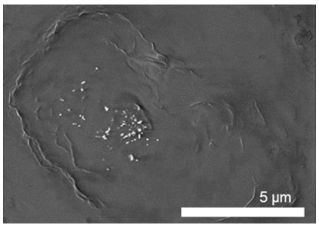 10k magnification	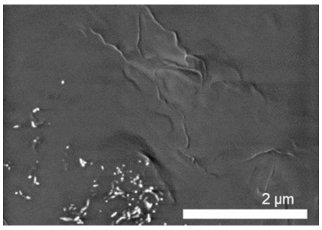 20k magnification	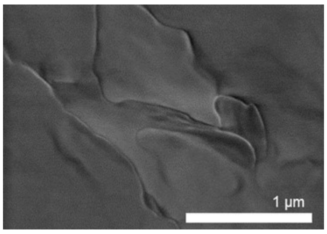 50k magnification
**PVA-MWCNT**	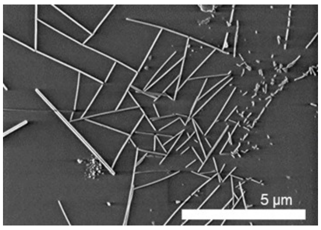 10k magnification	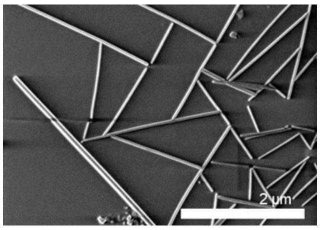 20k magnification	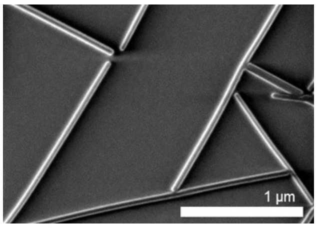 50k magnification
**PVA-rGO**	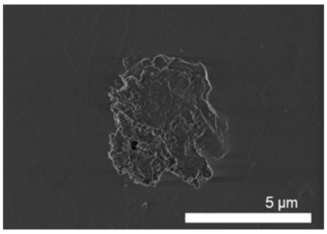 10k magnification	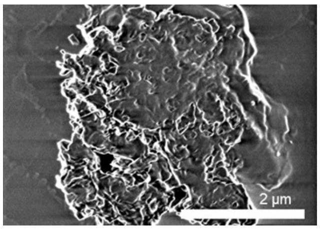 20k magnification	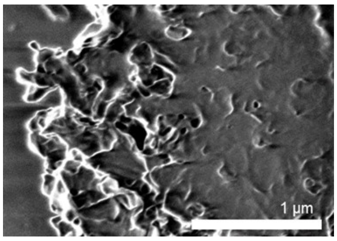 50k magnification

**PVA-C60**	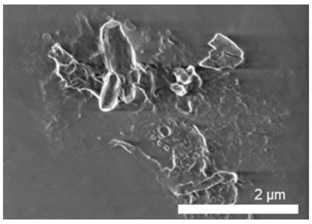 10k magnification	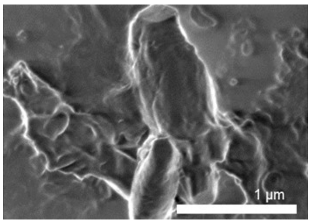 20k magnification	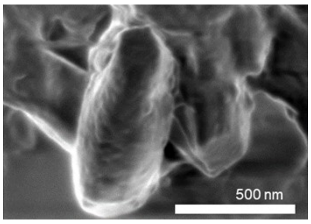 1 00k magnification
**PVA-SAC**	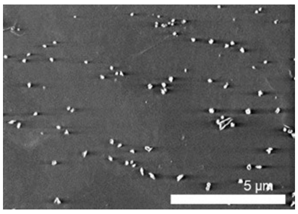 1 0k magnification	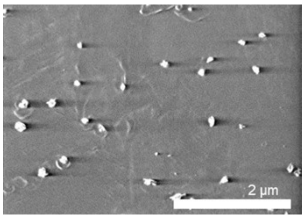 20k magnification	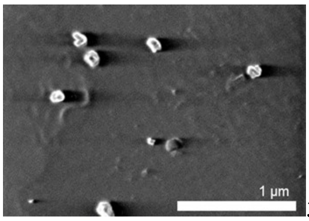 5 0k magnification

References

1 S. Mittal, R. K. Mondal, K. A. Dubey and Y. K. Bhardwaj, *J. Appl. Polym. Sci.*,2024, **141**. DOI:10.1002/app.54933.

2 K. Dash, B. Nayak and B. Prasad Sahoo, *Mater. Today Proc.*,2023. DOI:10.1016/j.matpr.2023.02.081.

3 Z. Liu, M. Muhammad, L. Cheng, E. Xie and W. Han, *Appl. Phys. Lett.*,2020, **117**. DOI:10.1063/5.0025001.

4 A. Ameli, P. U. Jung and C. B. Park, *Carbon N. Y.*, 2013, **60**, 379–391. DOI: 10.1016/j.carbon.2013.04.050.
